# Biostimulatory Effects of Amino Acids on Phenylalanine Ammonia Lyase, Capsaicin Synthase, and Peroxidase Activities in *Capsicum baccatum* L.

**DOI:** 10.3390/biology11050674

**Published:** 2022-04-27

**Authors:** Tilen Zamljen, Aljaz Medic, Metka Hudina, Robert Veberic, Ana Slatnar

**Affiliations:** Department of Agronomy, Biotechnical Faculty, University of Ljubljana, SI-1000 Ljubljana, Slovenia; aljaz.medic@bf.uni-lj.si (A.M.); metka.hudina@bf.uni-lj.si (M.H.); robert.veberic@bf.uni-lj.si (R.V.); ana.slatnar@bf.uni-lj.si (A.S.)

**Keywords:** biostimulant, capsaicin, enzyme, *trans*-cinnamic acid, time course

## Abstract

**Simple Summary:**

Very little is known about how biostimulants such as amino acids affect the enzymatic activity of peppers. The three crucial enzymes for the synthesis and degradation of capsaicinoids were studied in a 72 h time course. The activity of the two crucial enzymes for the synthesis of capsaicinoids increased in the first hour after foliar application and then gradually decreased to the baseline activity. Placenta exhibited higher enzyme activity and capsaicinoid content. Since capsaicinoids are widely consumed by humans, the use of various natural substances called biostimulants could improve the overall quality of peppers, which would mean high-yield and high-quality fruits.

**Abstract:**

Biostimulants are widely used in agriculture because they can improve fruit quality and quantity. Less is known about how biostimulants act over time in plants, in our case peppers, and how they affect the enzyme activity of important enzymes for capsaicinoid synthesis. The biostimulatory effects of amino acids on the activities of phenylalanine ammonia lyase (PAL), capsaicin synthase (CS), and peroxidase (POX) were investigated in the pericarp and placenta of the chili pepper *Capsicum baccatum* L. cv. “Bishop Crown” over 72 h of application. The PAL and CS activities significantly increased in the placenta after 1 h of biostimulant application, with significant increases of 130% and 16%, respectively. The POX activity remained unchanged over the full 72 h in the placenta but significantly increased after 48 h in the pericarp (+53%). Total capsaicinoids increased in the first hour of biostimulant application, by 4.30 g/kg FW in the placenta (19%) and by 0.94 g/kg FW in the pericarp (+56%). Biostimulant application also increased total and individual capsaicinoids after 48 h in the chili placenta and pericarp. With improved methods for enzymatic determination, we gained new insights into the responses of chilies to biostimulant amino acids.

## 1. Introduction

Chili peppers are known for their beneficial effects on human health due to their high vitamin and mineral content [1,2]. Metabolite accumulation in chili plants is influenced by many factors, such as climatic conditions and abiotic and biotic stresses [3,4,5].

The influence of these factors can be reduced by various agricultural measures, such as the use of biostimulants, which can reduce any stress and improve crop growth and yields [6]. The term “biostimulants” is used to describe plant hormones, amino acids, macro- and micronutrients, and other substances that have positive effects on plants [7]. Amino acids represent effective plant biostimulants, as they have important roles in many plant metabolic pathways [8]. As the synthesis of amino acids by plants themselves is energy consuming, the direct foliar application of amino acids to plants avoids this energy use for their synthesis. This allows the plants to divert the energy available to other energy-consuming activities, such as growth or the synthesis of metabolites such as capsaicinoids [9].

Capsaicinoids are the main pungent constituents of chilies and are found only in the genus *Capsicum*. The most abundant capsaicinoid is capsaicin, followed by dihydrocapsaicin (these two together account for 79–90% of the capsaicinoids) and nordihydrocapsaicin. Capsaicinoids are synthesized in the placenta of the fruit by the enzyme capsaicin synthase (CS) [10]. CS requires Mg^2+^, ATP, coenzyme A (CoA), and vanillylamine for the synthesis of capsaicinoids [11]. As reported by Mazourek et al. [12], other capsaicinoids are also synthesized by CS, and the variations in their structures occur along the synthetic pathway from valine to 8-methyl-6-nonenoic acid. The activities of such plant enzymes are also sensitive to abiotic and biotic stresses [13], as some of the molecules formed can act as stress reducers.

Capsaicin synthase is not the only important enzyme in the biosynthesis of capsaicinoids. Phenylalanine ammonia lyase (PAL) is also of particular importance, as it provides an important switch point between primary and secondary metabolism [14]. PAL catalyzes the process of nonoxidative deamination of phenylalanine to *trans*-cinnamic acid. Phenylpropanoids are derived from cinnamic acid and are then involved in the synthesis of other secondary metabolites [15], such as capsaicinoids.

As chilies mature, the degradation of capsaicinoids occurs. This is caused by the activity of peroxidase (POX), as reported by Díaz et al. [16]. Oxidation of capsaicinoids occurs at the vanillyl moiety because of its ease of oxidation [17]. High POX activity in chilies is usually seen in stressed plants and over-ripe fruit; it is thus indicative of degradation of the capsaicinoids [18,19]. This POX activity in sweet peppers can be reduced by the use of various biochemicals, such as methyl salicylate [19].

The present study investigated the direct metabolic responses of chilies to foliar biostimulant amino-acid treatments to determine how the synthesis of the capsaicinoids responded in the first 72 h after treatment. We examined the activities of PAL, CS, and POX in the post-treatment period of healthy chili plants to better understand the biostimulatory effects in the absence of other factors. The rare previous studies on the activities of these crucial enzymes for capsaicinoid synthesis (PAL, CS) and degradation (POX) have used spectrophotometric methods [10,11]. In the present study, we improved on these methods for the detection of the PAL and CS activities by HPLC–mass spectrometry, which provided greater credibility for the data obtained.

## 2. Materials and Methods

### 2.1. Materials

The following materials were used: Tris (hydroxymethyl aminomethane), 30% hydrogen peroxide, boric acid, and potassium dihydrogen phosphate (KPi) (all from Merck); ascorbic acid, sodium salt, and *trans*-cinnamic acid (both from Fluka); and ethylenediaminetetraacetic acid (EDTA), sodium tetraborate (borax), polyclar, sand, Sephadex G-25, o-dianisidine, L-phenylalanine, acetic acid, methanol, Tris-HCl, vanillylamine, ATP, MgCl_2_, 8-methyl-6-nonenoic acid, HCl, capsaicin, dihydrocapsaicin, nordihydrocapsaicin, formic acid, and acetonitrile (all from Sigma Aldrich).

### 2.2. Experimental Plants, Sampling, and Treatments

This study was conducted at the Biotechnical Faculty (46°3′4′′ N; 14°30′18′′ E) of the University of Ljubljana (Slovenia) from May 20 to September 30, 2020. The seeds of cultivar *Capsicum baccatum* L. cv. “Bishop Crown” was purchased from Austrosaat. The seeds were planted on February 15, and seedlings were then transplanted into plastic pots (φ 8 cm) filed with peat substrate (Neuhaus N3) when the first three true leaves developed. On May 20, ten plants of the chili pepper *Capsicum baccatum* L. cv. “Bishop Crown” were planted in a plastic greenhouse (spaced at 50 cm × 50 cm), with each plant equipped with a drip irrigation system through which irrigation water and fertilizer were administered. A water-soluble fertilizer was used (NPK fertilizer 16:8:32; Poly-Feed; with added micronutrients), as reported previously Zamljen et al. [20], five times during the plant growth, with the last dose being 10 days before the application of the biostimulant amino-acid solution (henceforth as “biostimulant”) on 10 September. The chili pepper plants were grown according to the integrated production guidelines of the Ministry of Agriculture, Forestry, and Food of Slovenia. The phytosanitary status was closely monitored during the seedling production and during the experiment itself so that no pests and disease could affect the experiment. The average greenhouse temperature from 20 May to 30 September was 23.3 °C, and the relative humidity was 79.5%.

The sampling of the chili fruit took place at 0 h (before application of the biostimulant) and then at 1, 3, 24, 48, and 72 h after the biostimulant application. Individually, each of the 10 plants represented a replicate within which three fruits were harvested at each timepoint (i.e., a total of 30 fruit harvested for each timepoint). The fruits were dissected, separating out the two fruit parts of the pericarp and placenta, to obtain a more detailed picture of how the biostimulant amino acids acted in these chili fruit.

The biostimulant amino-acid solution was from an animal protein hydrolysate and was used as supplied (Delfan Plus; Tradecorp International, Spain, Madrid). This contained 24% amino acids, 44.4% organic matter, and 10.8% total nitrogen, of which 6% was organic nitrogen, and 27.6% organic carbon, at pH 7.2. The aminogram of the amino acids provided by the manufacturer indicated: glycine (26.1%), glutamic acid (10.8%), alanine (10.3%), proline (10.3%), aspartic acid (7.2%), serine (7.1%), leucine (4.9%), hydroxyproline (3.5%), phenylalanine (3.3%), tyrosine (3.2%), arginine (2.8%), lysine (2.5%), valine (2.4%), threonine (2.2%), isoleucine (2.1%), methionine (0.9%), and histidine (0.4%).

When enough fruits were ripe on each plant, the first three fruits from each plant were picked for the control 0 h treatment. The biostimulant was then foliar applied to all of the plants at a dilution of 0.1% in water (1 mL of biostimulant in 1 L of water).

### 2.3. Sampling of Chilies

To compensate for the effects of different ripening stages, only fully ripe fruit that had the cultivar-specific red color, were firm to the touch, and had a glossy appearance were harvested, as reported previously by Zamljen et al. [21]. To compensate for further differences, the fruits were picked from different levels of the plants. The fruits were then separated into pericarp and placenta. A portion of the fruit tissue was frozen in liquid nitrogen, ground to a powder in a refrigerated mortar, and stored at −80 °C for the enzyme analysis. The other portion was stored at −20 °C for capsaicinoids extraction.

### 2.4. Phenylalanine Ammonia Lyase Assay

The PAL (EC 4.3.1.24) enzyme activity assay was performed as previously described by Phimchan et al. [10] and Cebulj et al. [22], with modifications. Here, 0.5 g fresh fruit tissue was mixed with 3 mL 0.1 M boric acid extraction buffer containing 0.4% sodium ascorbate (pH 8.5). The mixture was centrifuged at 12,000× *g* for 30 min (5810 R; Eppendorf). Gel chromatography columns (Sephadex G25 medium; Sigma-Aldrich, St. Louis, MI, USA) were conditioned with five successive washes with 1 mL extraction buffer. To remove low molecular weight compounds, the sample supernatants (500 µL) were added to the gel chromatography columns, which were then washed with 400 µL extraction buffer, and further used as crude extract.

The reaction mixture contained 110 µL extraction buffer, 80 µL crude extract, and 10 µL 10 mM L-phenylalanine. The blank sample contained 190 µL extraction buffer and 10 µL 10 mM L-phenylalanine. After addition of the L-phenylalanine, the samples were stirred on a vortex mixer (Top-Mix 94500; Bioblock Scientific, France, Strasbourg) and then incubated in a preheated oven at 37 °C for 1 h. After this incubation, the reaction was stopped by addition of 20 µL acetic acid and 400 µL MeOH. The samples were further stirred on a vortex mixer, transferred to vials, and stored at −20 °C until analysis.

Initial detection of the *trans*-cinnamic acid product of the PAL activity was by HPLC (UHPLC-PDA Dionex UltiMate 3000 system; Thermo Scientific, Waltham, MA, USA) at 280 nm, with the system settings based on Medic et al. [23]. The injection volume was 20 µL. A C18 column was used (Gemini; 150 × 4.60 mm, 3 μm; Phenomenex, Torrance, CA, USA) at 25 °C to separate the compounds. A discontinuous gradient was used with mobile phase A (0.1% formic acid with 3% acetonitrile in bidistilled water; *v*/*v*/*v*) and mobile phase B (0.1% formic acid with 3% bidistilled water in acetonitrile; *v*/*v*/*v*) at a flow rate of 0.6 mL/min. The gradient, washing, and reconditioning of the column between samples was as described by Wang et al. [24], as follows: 0–15 min, 5–20% B; 15–20 min, 20–30% B, 20–25 min, 30–50% B; 25–30 min, 50–90% B; 30–45 min, 90% B; 45–46 min, 90–5% B; 46–50 min, 5% B.

Identification of the *trans*-cinnamic acid as the measure of the PAL activity was performed using tandem mass spectrometry (LTQ XL 125; Thermo Scientific, Waltham, MA, USA) with heated electrospray ionization in negative ion mode. The scan of *trans*-cinnamic acid was at 280 nm, and its transition was [M − H]^−^ at *m*/*z* 147 with a typical fragmentation ion of *m*/*z* 103 (100), as previously reported by Ji et al. [25]. To confirm the correct identification of *trans*-cinnamic acid, an appropriate standard was used, according to the correct retention time and fragmentation. Chromatographic data of *trans*-cinnamic acid standard, sample, and blank and total ion chromatograms are given in Figure S1. The MS ions of *trans*-cinnamic acid are presented in Figure S2. The PAL activities were calculated according to Equation (1) [26] and are expressed as nmol/s × g.

(1) $$x=\frac{\mathrm{nmol}\;product\;\times \;60\;s\;\times \;V\;of\;extraction\;buffer\;\left(µL\right)}{incubation\;time\;\left(s\right)\;\times \;V\;of\;enzyme\;source\;\left(µL\right)\;\times \;mass\;of\;sample\;\left(g\right)}$$

### 2.5. Capsaicin Synthase Assay

The procedure for the CS (EC 2.3.2.35) activity was performed as reported by Phimchan et al. [10], with modifications. Here, 0.5 g of the freshly thawed sample (as a powder) was mixed with 3 mL 100 mM Tris-HCl buffer (pH 6.8). The samples were centrifuged at 10,000× *g* for 35 min (5810 R; Eppendorf, Germany, Hamburg), and the supernatant (as the CS source) was stored on ice. The reaction mixture contained 100 µL 0.4 M Tris-HCl buffer (pH 6.8), 10 µL 0.2 M vanillylamine, 5 µL 0.4 M ATP, 5 µL 0.4 M MgCl_2_, 5 µL 0.4 M 8-methyl-6-nonenoic acid, and 300 µL enzyme extract. The blank sample contained only the extraction buffer and the enzyme extract. The reaction mixture was mixed on a vortex mixer (Top-Mix 94500; Bioblock Scientific, France, Strasbourg). The incubation was performed in a preheated oven at 37 °C for 1.5 h, after which time the reaction was stopped with 100 µL 1 M HCl. The samples were then mixed again, and after addition of 500 µL MeOH, they were evaporated in vacuo at 50 °C. After addition of 1 mL 100% MeOH to the dried samples, they were shaken for 1 h on an orbital shaker (Unimax 1010; Heidolph, Germany, Schwabach). The samples were then stored in vials until analysis.

Capsaicin content was determined using an HPLC system (Dionex UltiMate 3000; Thermo Scientific, Waltham, MA, USA) with a quadrupole mass spectrometer (TSQ Quantum Access Max; Thermo Scientific, Waltham, MA, USA), as previously reported by Zamljen et al. [27]. Mobile phase A was 0.1% formic acid, 3% acetonitrile, and 97% bidistilled water, and mobile phase B was 0.1% formic acid, 3% bidistilled water, and 97% acetonitrile. The gradient for separation of the capsaicinoids was: 0 min, 40/60 (A/B); 1.5 min, 10/90 (A/B); 1.51 min, 40/60 (A/B); and 2.00 min, 40/60 (A/B), with a flow rate of 0.6 mL/min. The samples (20 μL) were injected onto the C18 column (Gemini; Phenomenex, Torrance, CA, USA) operated at 25 °C. The mass spectrometry was run with an electrospray ionization source in positive ion mode with the following settings: evaporator temperature, 300 °C; spray voltage, 4.5 kV; sheath gas, 50 arbitrary units (au); auxiliary gas, 20 au; and ion transfer capillary temperature, 320 °C. The range of the collected mass spectra was *m*/*z* 90 to 700. Argon was used as the collision gas. Selected reaction monitoring mode was used for data collection. The following transition was used to define capsaicin [28,29]: *m*/*z* 306 ► 137 (100), 170 (64), 182 (32) ► 122 (100), 109 (43), 91 (17). Capsaicin was additionally confirmed using an external standard. The peak width relative to resolution (i.e., full width at half maximum) was 0.1 μ for Q1 and 0.7 μ for Q3; collision gas pressure was 1.5 mTorr (1 Torr = 133 Pa); scan width was 0.4 *m*/*z*; and scan time was 0.1 s for each selected reaction monitoring transition. The CS activities were calculated according to Equation (1) [26] (see above) and are expressed as nmol/s × g.

### 2.6. Peroxidase Assay

The POX (EC 1.11.1.7) activity assay was performed as described by Cebulj et al. [22]. The extraction buffer contained 0.12% Tris, 0.2% EDTA, and 0.38% borax in bidistilled water. The freshly thawed samples were ground to a fine powder with 0.25 g polyclar and 0.25 g sand. The powder was then mixed with 3 mL extraction buffer. The samples were centrifuged at 10,000× *g* for 10 min. Meanwhile, the gel chromatography columns (Sephadex G25 medium; Sigma-Aldrich, St. Louis, MI, USA) were conditioned by addition of 1 mL 0.1 M KPi buffer (pH 6.5) five times. Four hundred microliters of the sample supernatants or enzyme source was pipetted onto the columns and allowed to stand until complete absorption. After absorption in the column, 400 µL 0.1 M KPi buffer was added to wash the sample into microcentrifuge tubes, which were placed in ice. The samples were then diluted as 370 µL 0.1 M KPi buffer and 30 µL sample. Prior to measurements, the H_2_O_2_–KPi buffer was prepared by mixing 1 mL 1% H_2_O_2_ and 99 mL 0.1 M KPi buffer (pH 6.5). The *o*-dianisidine mixture contained 1% *o*-dianisidine dissolved in MeOH, as substrate.

The reaction mixture contained 1050 µL H_2_O_2_–KPi buffer, 50 µL diluted samples and 10 µL *o*-dianisidine. The blank sample contained 1100 µL H_2_O_2_–KPi buffer and 10 µL *o*-dianisidine. Measurements were performed on a UV–vis spectrophotometer (Genesys 10S; Thermo Scientific, Waltham, MA, USA) with continuous measurements at 460 nm every 5 s for 15 min. The POX activities were calculated according to Equation (2) [26] and are expressed as ΔA/min.

(2) $$x=\frac{two\;highest\;differeneces\;among\;absorbtions\;between\;each\;minute}{2}$$

### 2.7. Extraction and Analysis of Capsaicinoids

The extraction procedure for the capsaicinoids was carried out as reported by Zamljen et al. [27] with minor modifications. The pericarp and placenta fruit tissue was thawed and ground to a powder in a refrigerated mortar, and 0.3 g of this powder was extracted with 3 mL 100% MeOH in a cooled ultrasonic bath (0 °C) for 1 h. The samples were then centrifuged at 8000× *g* for 5 min, filtered through 25 µm polyamide filters (Chromafil AO 45/25; Macherey-Nagel, Dueren, Germany), and then stored in vials at −20 °C.

Capsaicin, dihydrocapsaicin, nordihydrocapsaicin, homocapsaicin, and homodihydrocapsaicin were quantified using an HPLC system (Dionex UltiMate 3000; Thermo Scientific, Waltham, MA, USA) combined with a quadrupole mass spectrometer (TSQ Quantum Access Max Thermo Scientific, Waltham, MA, USA). A C18 column was used (Gemini; Phenomenex, Torrance, CA, USA), with the other settings as described previously by Zamljen et al. [27]. The data are expressed as g/kg FW.

### 2.8. Statistical Analysis

Statistical analysis was performed using the R statistical environment [30]. The data are expressed as means ±standard error (SE). For determination of statistically significant differences between the samples, one-way analysis of variance (ANOVA) was used with Tukey’s tests. Statistical means at a 95% confidence level were calculated to determine the significance of the differences.

## 3. Results

### 3.1. Enzyme Activities and Total Capsaicinoids Contents

**Figure 1:**
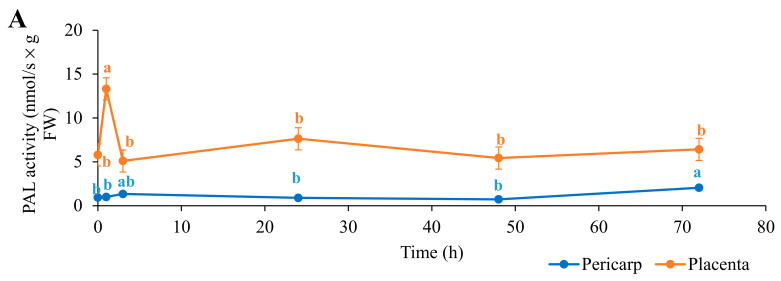

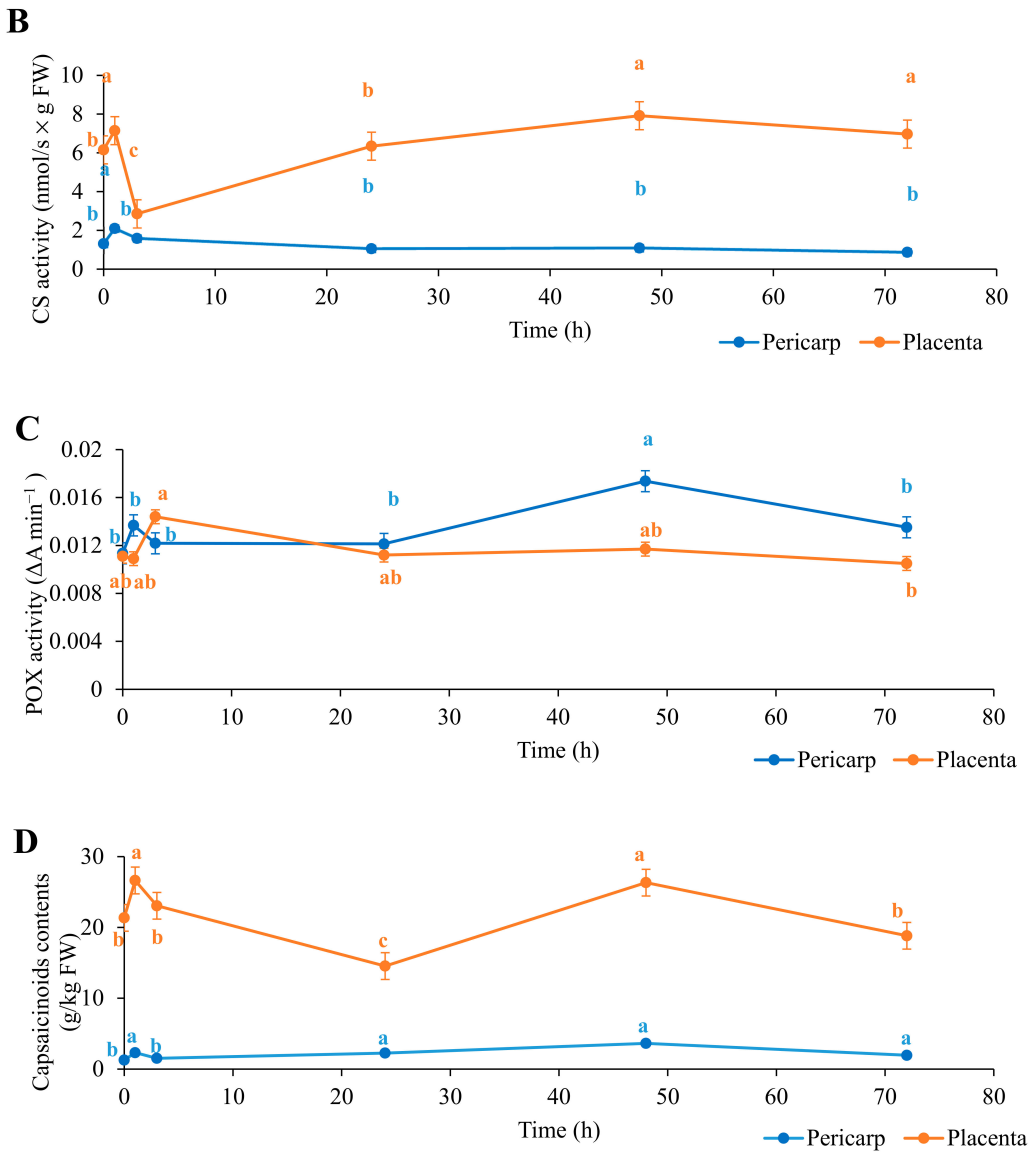
Time courses following biostimulant application for the absolute enzyme activities for phenylalanine ammonia lyase (PAL; (**A**)), capsaicin synthase (CS; (**B**)), and peroxidase (POX; (**C**)) and for the total capsaicinoid content (**D**) in pericarp and placenta of the chili fruit. Data are means ±standard error (n = 5). Data with different lowercase letters are significantly different (ANOVA; Tukey’s tests).

The absolute activities of PAL, CS, and POX and the total capsaicinoid contents following biostimulant application are shown in Figure 1.

The placenta of the chilies initially showed higher PAL and CS activities than the pericarp, by some fourfold and sixfold, respectively. The PAL and CS activities in the placenta then significantly increased after the first hour of biostimulant application, for PAL by 7.54 nmol/s × g FW (130%) and for CS by 1.00 nmol/s × g FW (16%). Conversely, the POX activity was similar for both parts of the chilies. For the placenta, there were no significant changes throughout the full 72 h, while for the pericarp, there was a small, but significant, peak at 48 h after biostimulant application before returning to the original level. Following the large peak in PAL activity at 1 h in the placenta, PAL activity decreased to the initial levels before biostimulant application and remained unchanged through 72 h. Interestingly, the CS activity in the placenta decreased below the initial level after 3 h, to reach its lowest level of 2.85 nmol/s × g FW (54% lower), before returning again from 24 h to 72 h to similar, if slightly higher, levels as those before biostimulant application.

For the PAL activity in the pericarp, the only change seen was a small, but significant, increase at 72 h of 1.12 nmol/s × g FW (+20%) compared with before biostimulant application. Instead, the changes in the CS activity in the pericarp were similar to those in the placenta, with an initial significant increase (+60%) after the first hour of biostimulant application before returning to the initial levels from 3 h to 72 h. In parallel with the higher initial absolute PAL and CS activities in the placenta than in the pericarp, the total capsaicinoid contents were initially nearly 15-fold higher in the placenta (Figure 1). At the same time, the total capsaicinoid content significantly increased in the first hour after biostimulant application for both the placenta and pericarp, by 19% and 56%, respectively. In the placenta, the total capsaicinoid content then decreased to its lowest level at 24 h, at 16.65 g/kg FW, and then to its highest level at 48 h, at 30.78 g/kg FW; this then returned to the initial level after 72 h. Following the initial increase at 1 h in the pericarp, the total capsaicinoid content decreased to similar levels as those before biostimulant application and then showed a small, but significant, increase for 24 h to 72 h to levels similar to those at the peak seen at 1 h.

### 3.2. Individual Capsaicinoids after Biostimulant Application

Capsaicin and dihydrocapsaicin accounted for >90% of all of the five capsaicinoids detected in these chili fruit tissues (Table 1). Biostimulant application affected most of the individual capsaicinoid levels, except for those of homodihydrocapsaicin (placenta and pericarp) and nordihydrocapsaicin (placenta). Capsaicin significantly increased in both fruit parts by the first hour of biostimulant application and then decreased through 3 h. Interestingly, capsaicin significantly increased in the pericarp after 24 h and also in the placenta after 48 h. In the pericarp, dihydrocapsaicin, nordihydrocapsaicin, and homocapsaicin significantly increased in the first hour and then decreased at 3 h of biostimulant application. From 24 h to 72 h, the levels of these capsaicinoids in the pericarp then increased again and remained significantly higher than their initial levels before biostimulant application. In the placenta, only homocapsaicin also showed a significant initial increase at 1 h, with few further changes seen, and with all of the individual capsaicinoids returning to the levels before biostimulant application by 72 h.

## 4. Discussion

Potential biostimulant amino acids were applied to the foliage of healthy chili plants (*C. baccatum* L. cv. “Bishop Crown”) to determine the enzymatic (PAL, CS, POX) and metabolic (capsaicinoids) responses in the fruit placenta and pericarp over time. The placenta contained more capsaicinoids and had higher PAL and CS activities than the pericarp, which was consistent with a study by Cervantes-Hernández et al. [30]. In particular, the biostimulant increased the PAL and CS activities in the placenta, which represents the main site of capsaicinoid synthesis [31], after the first hour of application. In the pericarp, although it was initially lower than in the placenta, the CS activity increased more proportionally in the first hour; consequently, the levels of the capsaicinoids also increased. Interestingly, after 3 h of biostimulant, the PAL and CS activities decreased, although no significant changes were seen for POX. In the placenta, the corresponding decrease in the capsaicinoids was then gradual, to reach the lowest level after 24 h. In the pericarp, however, the total capsaicinoid content decreased after 3 h of biostimulant but then increased to higher levels than before biostimulant application.

The major nitrogen transporters in plants are glutamate, glutamine, aspartate, and asparagine, which also act as major sources of accumulated nitrogen in leaves and other plant tissues [9]. Foliar application of these biostimulant amino acids, which are high in nitrogen, stimulates nitrogen translocation and accumulation, as previously reported in strawberry [8]. Indeed, the glutamate biosynthesis pathway (i.e., the GS–GOGAT pathway) is important for nitrogen assimilation [32]. The process of transferring amine from glutamate to 2-oxo precursors of serine, aspartate, alanine, valine, leucine, isoleucine, phenylalanine, and tyrosine is catalyzed by transaminases [32]. This process produces two important amino acids, valine and phenylalanine, which are required for the synthesis of capsaicinoids [31]. Natural biostimulants such as humic acids or amino acids additionally affect carbon metabolism enzymes involved in glycolysis, the Krebs cycle, and nitrate assimilation [33], which are important for plant secondary metabolism and enzyme activities.

Phenylalanine ammonia lyase is important for the synthesis of many secondary metabolites, such as phenols and capsaicinoids [34]. PAL converts phenylalanine into *trans*-cinnamic acid, which is then further processed and incorporated into other synthetic pathways [15]. In addition, CS uses vanillylamine and 8-methyl-6-nonenoic acid to produce capsaicinoids, which are derived from valine and phenylalanine after several steps. With the foliar application of these amino acids, we provided the plant cells (and thus these enzymes) with additional nitrogen, carbon, and other substances. When these enzymes had enough substrates available to drive their activities, they produced more metabolites, which was shown here for the placenta PAL and CS activities and the pericarp CS activity in the first hour after biostimulant application. As Ruan and Gerendás [35] reported, nitrogen applied through the leaf is taken up in the first 48 h after application. This was particularly noted here in the activity of the placenta CS, which was higher after 48 h than before biostimulant application. After 3 h of biostimulant, we observed a decrease in CS activity, although with no significant change in POX activity. Increased POX activity was reported by Oraghi Ardebili et al. [36]; this was mainly dependent on the amino-acid concentration. Increased POX activity after biostimulant application was also reported by Alturki et al. [37] for tomato plants treated with amino acids and other biostimulants (see also Drobek et al. [38]). Reactive oxygen species (ROS), such as OH, O_2_^−^, and H_2_O_2_, are harmful to cells, as they cause oxidative stress. POX is responsible for reducing or reversing these negative effects [39]. Although the biostimulant amino acids here did not significantly increase POX activity, there may well have been decreased oxidative stress; however, in chilies, POX is also responsible for degradation of capsaicinoids [10], which was indeed seen 24 h after biostimulant application.

## 5. Conclusions

Using improved methods to determine PAL and CS activities (i.e., combined HPLC and mass spectrometry), the present study provided meaningful insight into how amino acids biostimulant affected capsaicinoid biosynthesis and contents in the chili *C. baccatum* cv. “Bishop Crown” pericarp and placenta over the first 72 h of their application. In addition, POX activity was determined spectrophotometrically. We observed strong enzymatic responses in the first hour after biostimulant application, particularly for PAL and CS in the placenta, confirming the beneficial effect of these amino acids on chili metabolism. This application of biostimulant amino acids had positive effects on the total capsaicinoid contents and on the individual capsaicinoids in the placenta and pericarp of these chili fruit after 48 h. The responses of the placenta to biostimulant application were stronger than those of the pericarp, as the main tissue of synthesis and enzymatic activity in chili fruit is the placenta. With these improved, more accurate methods for enzymatic determination in chilies, we gained new insight into the responses of unstressed chili plants to biostimulants, thus laying the groundwork for further studies that will incorporate abiotic and biotic stressors.

## Figures and Tables

**Table 1:** Individual capsaicinoids identified in the chili fruit pericarp and placenta before and after biostimulant application (mean ± SE).

Tissue	Biostimulant	Capsaicinoids (g/kg FW)				
	(h)	Capsaicin	Dihydro-Capsaicin	Nordihydro-Capsaicin	Homo-Capsaicin	Homodihydro-Capsaicin
Pericarp	0	1.34 ± 0.22 c	0.28 ± 0.05 b	0.02 ± 0.00 b	0.02 ± 0.00 c	0.01 ± 0.00 a
	1	2.03 ± 0.25 a	0.45 ± 0.05 a	0.04 ± 0.00 a	0.07 ± 0.00 a	0.01 ± 0.00 a
	3	1.19 ± 0.11 c	0.26 ± 0.02 b	0.02 ± 0.00 b	0.02 ± 0.00 c	0.01 ± 0.00 a
	24	2.21 ± 0.18 a	0.46 ± 0.04 a	0.05 ± 0.00 a	0.05 ± 0.00 a	0.01 ± 0.00 a
	48	1.71 ± 0.03 b	0.42 ± 0.01 a	0.04 ± 0.00 a	0.06 ± 0.00 a	0.01 ± 0.00 a
	72	1.52 ± 0.19 b	0.39 ± 0.01 a	0.04 ± 0.00 a	0.04 ± 0.00 b	0.01 ± 0.00 a
Placenta	0	16.85 ± 0.95 b	4.71 ± 0.37 a	0.27 ± 0.03 a	0.73 ± 0.10 b	0.64 ± 0.05 a
	1	19.64 ± 0.36 a	5.44 ± 0.13 a	0.37 ± 0.00 a	1.35 ± 0.08 a	0.70 ± 0.00 a
	3	15.17 ± 0.10 b	4.30 ± 0.12 a	0.25 ± 0.02 a	0.82 ± 0.01 b	0.56 ± 0.03 a
	24	11.94 ± 0.32 c	3.39 ± 0.05 b	0.22 ± 0.00 a	0.61 ± 0.01 b	0.46 ± 0.00 a
	48	22.71 ± 2.79 a	5.66 ± 0.79 a	0.34 ± 0.06 a	1.40 ± 0.19 a	0.65 ± 0.12 a
	72	14.40 ± 1.05 b	3.94 ± 0.37 a	0.25 ± 0.03 a	0.72 ± 0.09 b	0.55 ± 0.05 a

Data are means ±standard error (n = 5). Data with different lowercase letters are significantly different (ANOVA; Tukey’s tests).

## Data Availability

The data presented in this study are available on request from the corresponding author. The data are not publicly available because of privacy.

## Supplementary Materials

The following supporting information can be downloaded at: https://www.mdpi.com/article/10.3390/biology11050674/s1, Figure S1: Chromatographic data of *trans*-cinnamic acid standard (**A**), *trans*-cinnamic acid sample (**B**), blank sample (**C**), and the total ion chromatogram showing *trans*-cinnamic acid (**D**).; Figure S2: *Trans*-cinnamic acid MS ions, MS^n^ 147 (**A**) and MS ^2^ 103 (**B**).
