# Supplementary material for: Biostimulatory Effects of Amino Acids on Phenylalanine Ammonia Lyase, Capsaicin Synthase, and Peroxidase Activities in Capsicum baccatum L
Source: Biology (Basel). 2022 Apr 27;11(5):674. doi: 10.3390/biology11050674 (PMC9138247; doi:10.3390/biology11050674)
Supplement: Supplementary file 1 [file biology-11-00674-s001.zip › biology-1654798-supplementary.pdf]

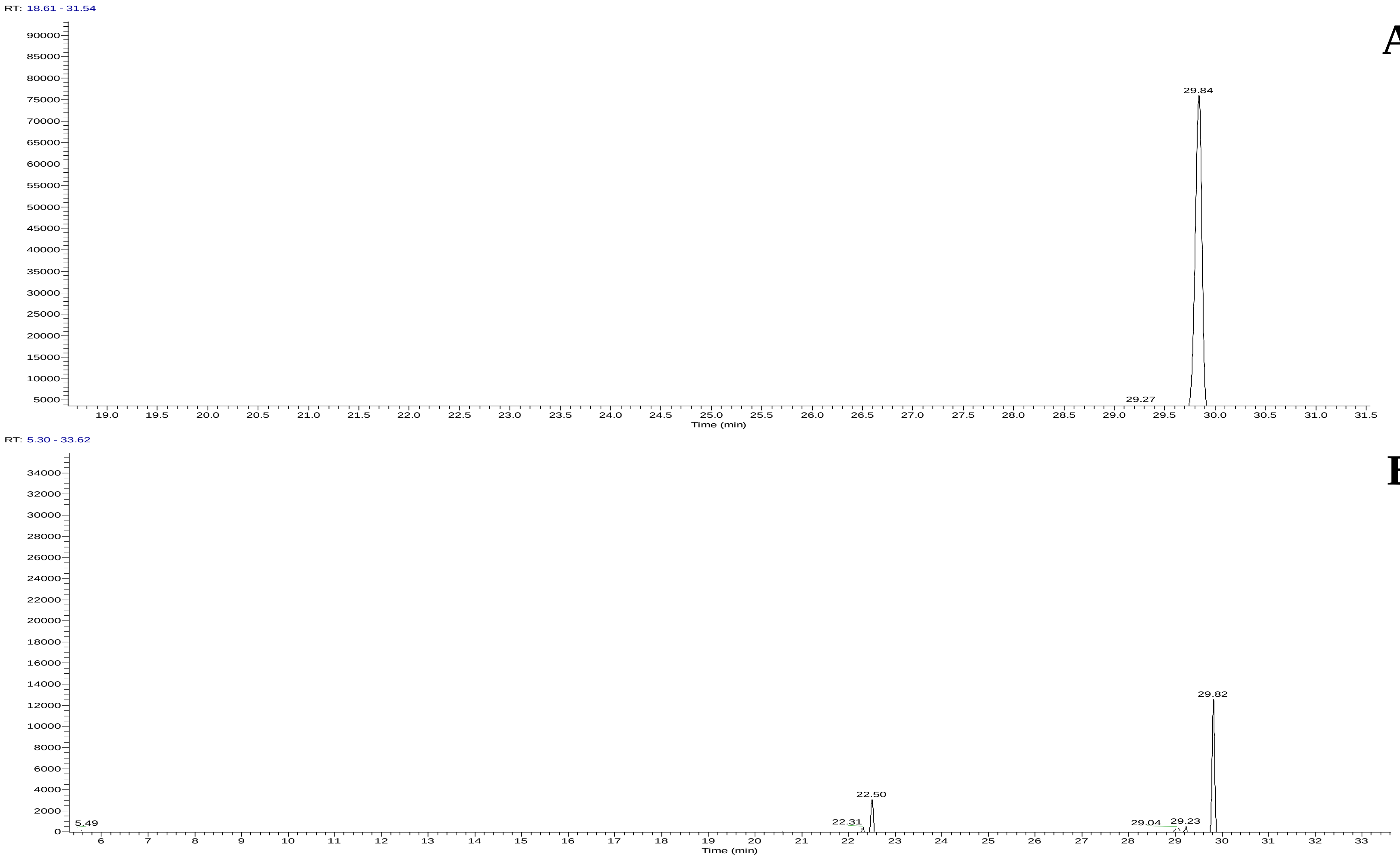

RT: 18.61 - 31.54
A
29.84
29.27
Time (min)
RT: 5.30 - 33.62
B
29.82
22.50
22.31
29.04
29.23
5.49
Time (min)

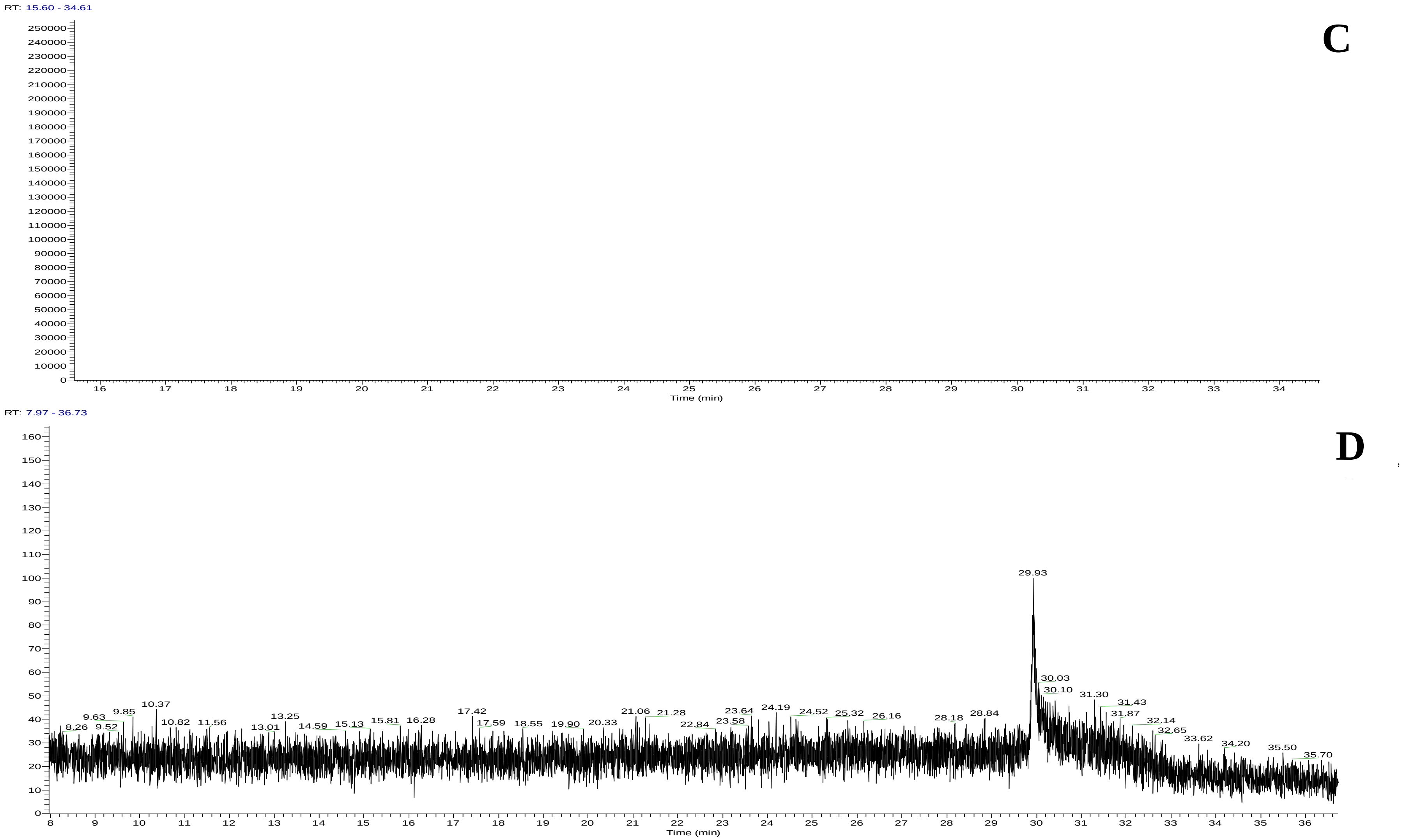

Figure S1. Chromatographic data of *trans*-cinnamic acid standard (**A**), *trans*-cinnamic acid sample (**B**), blank sample (**C**), and the total ion chromatogram showing *trans*-cinnamic acid (**D**).

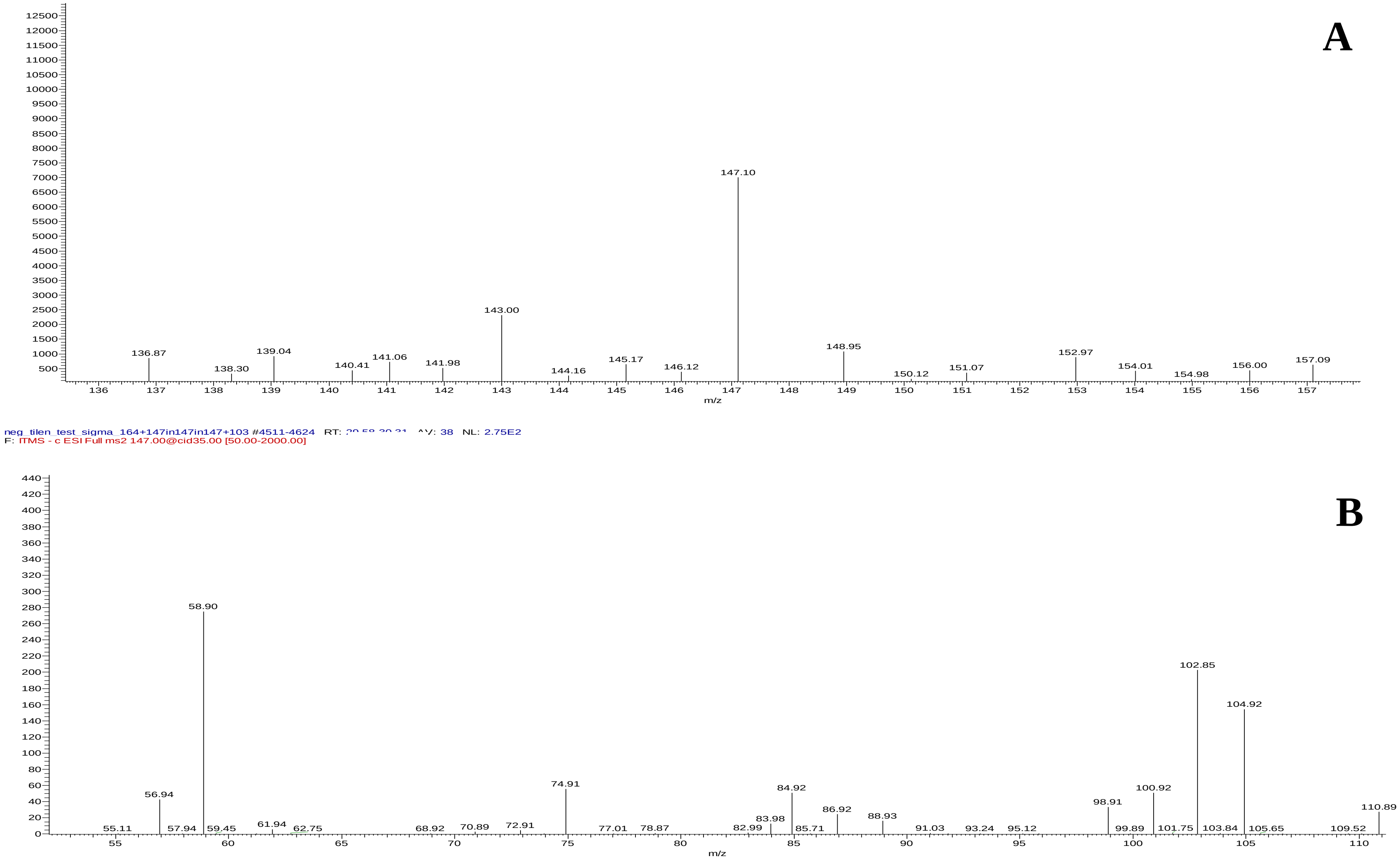

Figure S2. *Trans*-cinnamic acid MS ions, MS^n 147 (**A**) and MS^2 103 (**B**).
